# Usability Testing and Piloting of the Mums Step It Up Program - A Team-Based Social Networking Physical Activity Intervention for Women with Young Children

**DOI:** 10.1371/journal.pone.0108842

**Published:** 2014-10-01

**Authors:** Jocelyn Kernot, Tim Olds, Lucy K. Lewis, Carol Maher

**Affiliations:** Health and Use of Time (HUT) Group, Sansom Institute for Health Research, School of Health Sciences, University of South Australia, Adelaide, Australia; National Center of Neurology and Psychiatry, Japan

## Abstract

**Background:**

Women’s physical activity levels decline during their transition to parenthood. Facebook is widely used by Australian mothers and provides the opportunity to target social networks in order to maintain and increase physical activity.

**Method:**

This mixed method study aimed to pilot and assess the usability of the Mums Step It Up Facebook app, a new team-based physical activity intervention for mothers with young children. A purposive sample of five “Captain” women with young children, were recruited through personal contacts. These women used the app to recruit 3–7 Facebook friends (with children under 5) to join their respective teams (total n = 25). The app encourages women to take 10,000 steps a day measured by a pedometer. Women used the app for 28 days to log steps, interact with team mates and monitor progress. Physical activity was assessed at two time points (baseline and final week) using the Active Australia Survey. Usability testing with the five “Captain” women took place over two one hour face-to-face sessions. A questionnaire seeking feedback on the app was completed at time point two.

**Results:**

Participants’ total physical activity increased by an average of 177 minutes per week (*p* = 0.01). The complexity of the team forming process and issues using the Facebook environment, where a variety of devices and software platforms are used, was highlighted.

**Discussion:**

A team-based Facebook app shows considerable promise for the recruitment and retention of participants to a social network-based physical activity intervention. A randomised controlled trial to further evaluate the effectiveness of the intervention is warranted.

## Introduction

Insufficient physical activity is linked to many chronic diseases such as cardiovascular disease, diabetes, cancer and osteoporosis [1]. Furthermore, inactive people miss out on the benefits of regular physical activity such as the positive effects on mood, energy, wellbeing, cholesterol levels, insulin sensitivity and aerobic fitness [2], [3]. Women with young children are particularly at risk of physical inactivity [4], [5].

Online social networks offer considerable potential for the delivery of low cost, mass reach health-based interventions [6]. Facebook has 1.11 billion users world-wide, with over 50 per cent of users visiting the site daily [7]. In Australia, social networking accounts for one in every five minutes that Australians spend online [8], with Facebook attracting 13.2 million Australians each month [9] and nine million Australians each day [10].

Women’s use of Facebook increases during their transition to parenthood [11]. Eighty per cent of Australian mothers use Facebook daily, with mums identifying Facebook as their primary means of keeping in touch with friends and family [11], [12]. Key features of Facebook, such as its 24-hour day availability, flexibility and saliency, suggest that it may be a useful platform for the delivery of health interventions for this population.

A recent systematic review identified four studies which have attempted to use Facebook to alter physical activity behaviours [13]. Three of these studies used a Facebook community group with a discussion board, and produced modest results [14], [15], [16]. A strength of Facebook is the ability to recruit users and deliver an intervention via online social networks. One pilot study reporting a team-based physical activity intervention showed promising results, however the intervention was short in duration (5 days) and the sample size was small (n = 10) [17]. In recent times there has been increasing awareness that health issues and behaviours can “spread” through social networks. Recent work by Leahey et al [18], [19] suggests that teams can mediate the effect of health interventions.

In light of such findings, we created a Facebook app, titled “Mums Step It Up”, which is used to deliver a team based physical activity intervention for mothers with young children. This study aimed to determine the usability of the Mums Step It Up Facebook app. The primary aims were to 1) scrutinise: the ease of navigation, appropriateness of language, instructions, interest and overall appeal of the Mums Step It Up app; and 2) evaluate whether the app could successfully allow teams to self-assemble via existing social networks; The secondary aims were to: 3) examine the effectiveness of the app for changing physical activity behaviour; and 4) evaluate user retention and engagement with the app.

## Method

This study meets PLOS ONE guidelines for new methods software and databases.

### 2.1 Ethics

This study was approved by the University of South Australia Human Research Ethics Committee (protocol number: 00000030420). Participants consented to participate via an online registration form which they completed as part of the Mums Step It Up app. Data were collected between April and June 2013.

### 2.2 Intervention

The Mums Step It Up Facebook app (http://apps.facebook.com/fbexper) is designed to encourage women to reach the recommended public health physical activity goal of 10,000 steps per day [20], [21], [22]. Women used the app for 28 days with the cumulative goal being 280,000 steps. They participated in teams of four to eight friends and measured their daily step count with a pedometer.

The Mums Step It Up app is based on the Theory of Planned Behaviour [23], [24] and Fun Theory [25]. The Theory of Planned Behaviour proposes that an individual’s decision to go ahead with a particular behaviour is influenced by three factors: attitude, subjective norms and perceived behavioural control [23], [24]. The app attempts to address each of these factors by: customising features to ensure they are relevant and appealing to mothers with young children (attitudes, perceived behavioural control); use of teams for peer encouragement and support (subjective norms); and setting small achievable goals (daily step count) which are recorded and contribute to a long term/overall goal (280,000 steps) (attitude, perceived behavioural control).

**Figure 1:**
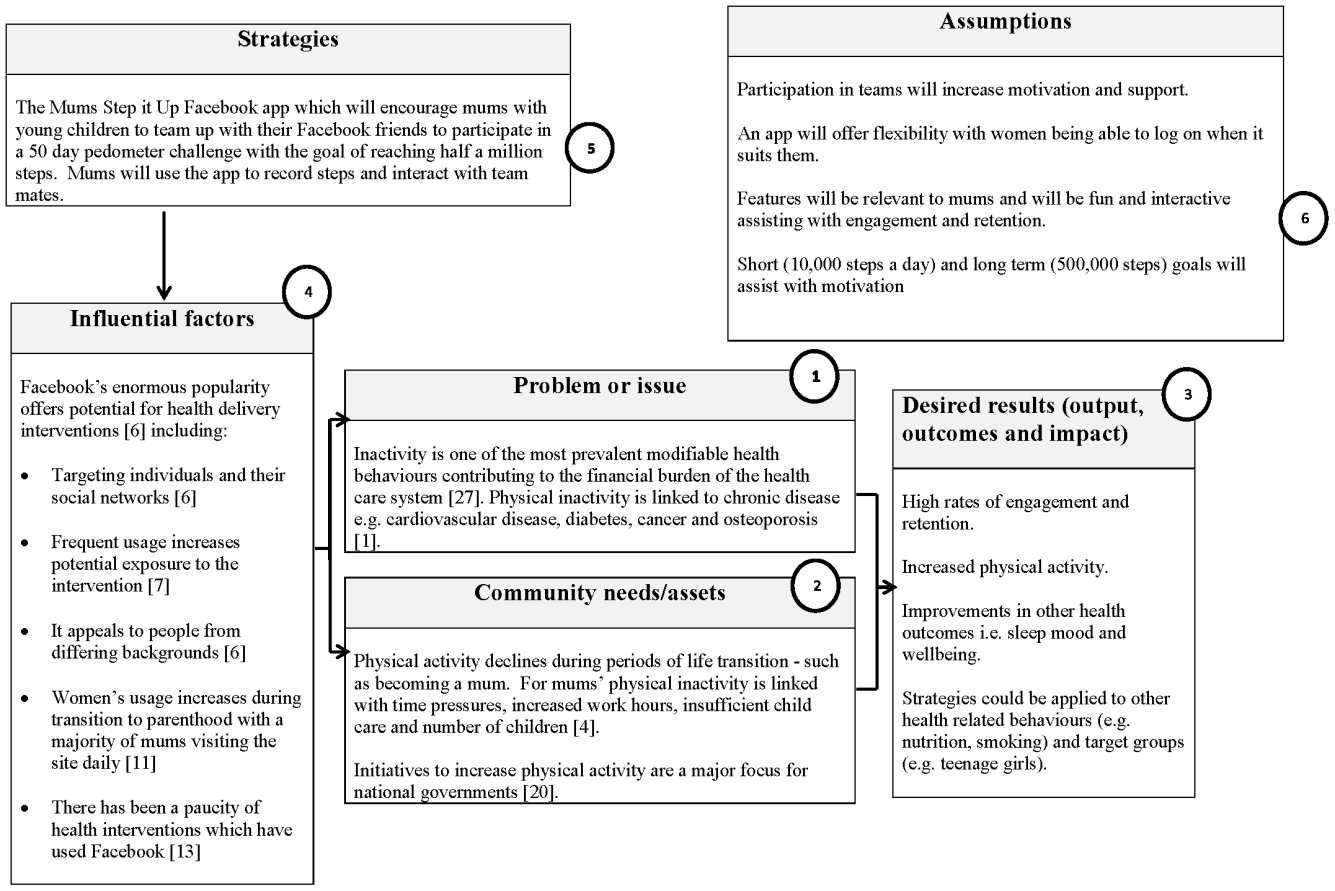
Flow chart of the Mums Step It Up program theory.

**Figure 2:**
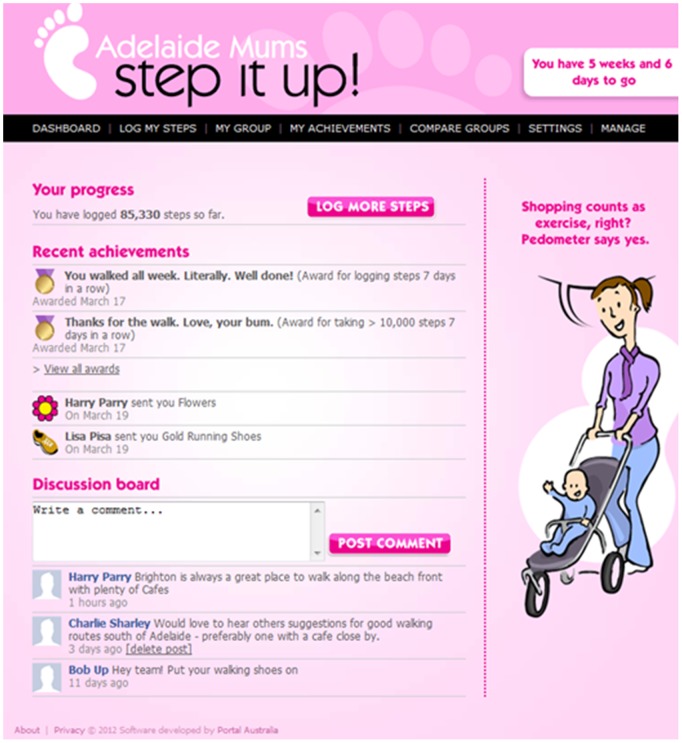
Screen shot of the dashboard of the Mums Step It Up app. The Dashboard provides links to all of the other pages.

Fun theory is not a true theory as it is not based on rigorous research, but rather, a philosophical campaign which advocates that if routine activities are adapted to be fun, people are more likely to be motivated to do them [25]. The Mums Step It Up app has been designed to include a number of fun and interactive features; a comedian has assisted with writing daily tips for increasing physical activity, and awards which participants can unlock based upon step count, login and team achievements. Team mates can also send each other virtual gifts for encouragement. Additional feedback is provided regarding step count achievements via a team tally board, graphs and statistics on hours of life gained, fat burned, carbon emissions and transport costs saved. Participants receive weekly emails detailing their progress and reminding them to log their steps. Figure 1 includes a flow chart (based on the Kellogg Foundation Logic Development Guide [26]) of the program theory detailing the rationale behind the Mums Step it Up app. Figure 2 shows the app dashboard.

### 2.3 Participants

To be eligible to participate, women had to 1) have a child under five years old, 2) be current Facebook users, 3) be able to read and understand English and 4) live in greater metropolitan Adelaide (Australian city of approximately one million people). Women were excluded if they had a medical condition that prevented them from participating in a walking program or if they were pregnant or planning on becoming pregnant in the next three months.

Women were recruited in two stages. Firstly, a purposive sample of five women (“Captains”) was recruited through personal contacts. The second stage involved a snowballing recruitment method in which “Captains” used the team formation processes built into the Facebook app to recruit three to seven of their eligible Facebook friends each to join their teams (“Team Members”).

### 2.4 Procedure

“Captain” women attended two one-on-one sessions with the principal investigator (JK) where they were observed using the Facebook app. The sessions took place at their homes or at the University of South Australia, with approximately five weeks between sessions. “Team Members” had no face-to-face contact with the principal investigator; rather they participated via the Facebook app, with email and phone contact as needed. Following registration, all participants received a pedometer (NL-1000) in the mail.

This procedure was chosen to test the app as it is intended to be used (i.e. for recruitment/team formation and as a physical activity intervention). The detailed procedures related to each of the study aims are described below.

#### Usability issues

Substantial alpha testing of the Facebook app by the researchers and software developers occurred in the five months prior, with the aim of identifying and rectifying technical issues before the commencement of the study.

Usability was scrutinised in two ways, 1) a feedback questionnaire completed by all participants in the last week of the Mums Step It Up challenge, and 2) by direct observation of “Captains” during the two face-to-face sessions. The feedback questionnaire (File S1) comprised 23 items designed to gather participants’ opinions regarding the app including: ease of use, appeal to the target audience, interest and impact of the key features, and suggestions for improvement.

At each face-to-face session, “Captains” were observed using the app, and asked to undertake all key tasks, such as sending invitations, logging daily steps, monitoring their own and their team’s achievements and interacting with team mates via the discussion board. Participants were asked to “think aloud” (i.e. verbally describe thought processes and decision making). The principal investigator observed participants and completed a checklist which detailed each of the steps, whether they were completed successfully and any questions asked. Observations were also made on body language, such as signs of confusion or frustration, and the time taken to complete each step.

#### Team forming process

**Figure 3:**
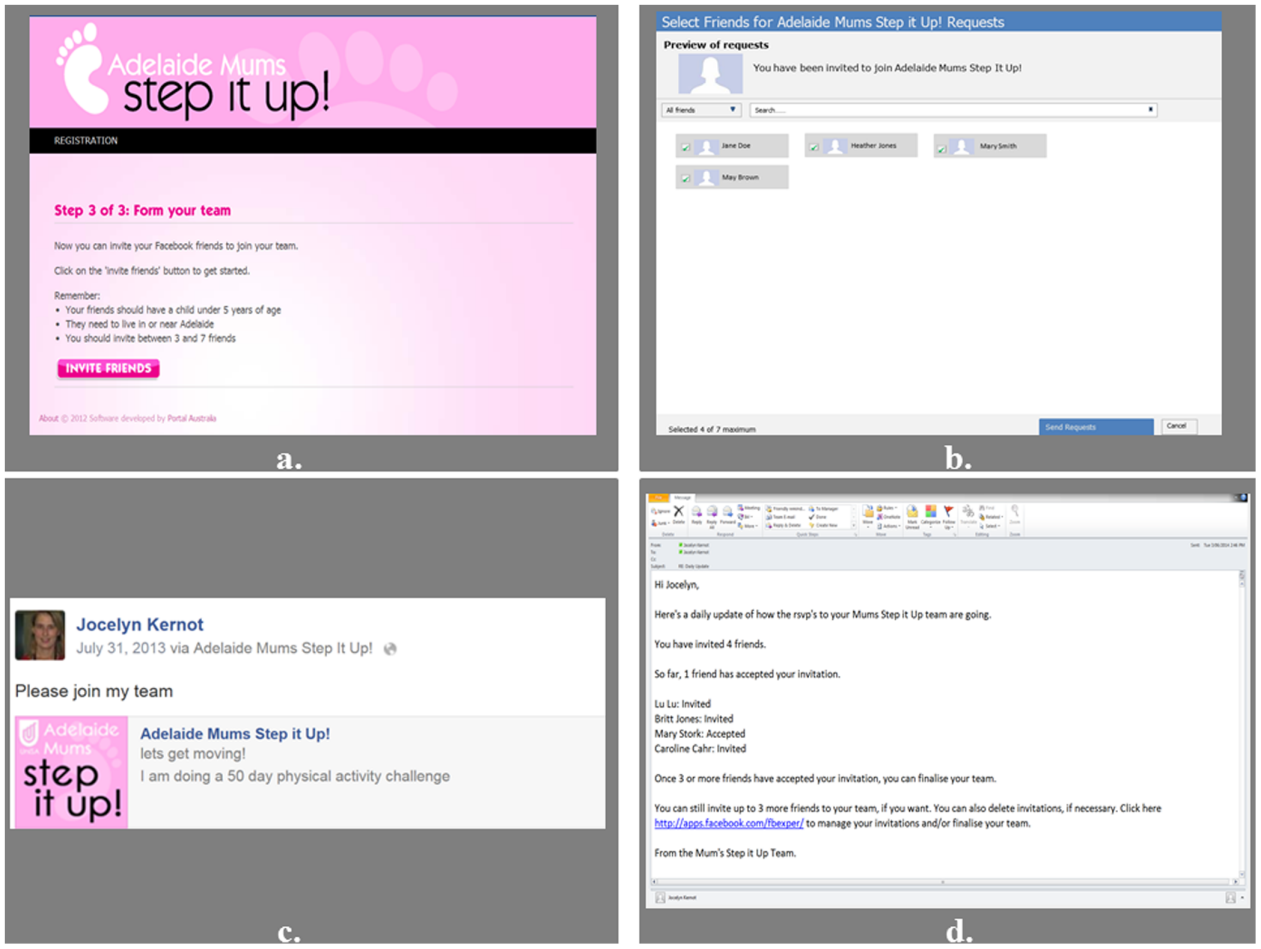
Inviting Facebook friends to join a team using the Mums Step It Up app. a) Invite Friends Page. b) When participants click on the “invite friends” button, a pop up appears listing their Facebook friends. They select 3–7 friends that they would like to send an invitation to. c) When they click on the “send request” button, another pop up appears which allows them to send a personalised message to post on their friends’ Facebook wall. d)”Captain” women receive daily emails updating them on their friends’ responses to their invitations.

The Mums Step It Up app includes a team building feature. During the first face-to-face session the “Captains” sent invitations to eligible existing Facebook friends. This was completed via the app’s “invite friends page” (Figure 3), which includes a pop up listing of all their Facebook friends. Once they selected friends to invite, they were able to post the invitation with an optional personalised message on to their friends’ Facebook wall. When their friends clicked on the invitation it directed them to the app, where they could watch a video summarising the study information, and either decline or accept by completing an online registration and consent form.

In the ensuing days “Captains” received daily emails informing them who had accepted the invitation to join their team. “Captains” could go back to the app at any stage to invite more friends or delete an invitation if one of their friends had indicated that they did not want to be involved, or hadn’t responded. Once the “Captain” had recruited at least three friends, they could finalise their team (via the app), or wait for more friends to respond.

#### Effectiveness for increasing physical activity

Physical activity was assessed at two time points: 1) baseline, immediately prior to commencing the Mums Step It Up program, and 2) in the final week of the intervention. Physical activity was measured using the Active Australia Survey [27] which invites participants to recall their physical activities over the previous seven days. The survey includes questions relating to the frequency and duration of: walking (for exercise, recreation or transport); vigorous physical activities (such as jogging, cycling, aerobics and competitive sport); and moderate physical activity (such as gentle swimming, tennis and golf). The Active Australia Survey has been shown to have moderate reliability (reliability coefficient for frequency/duration ranged from 0.56 to 0.64 for each of the physical activity domains) [28] and moderate validity when compared with weekly pedometer step counts (rho = 0.43) and accelerometery (rho = 0.52) [28].

#### User retention and engagement

User retention and engagement with the app were determined via usage data (steps logged, daily logins, number of interactions with team mates) recorded by the app.

### 2.5 Data analysis

Participant characteristics (age, education, number of children, work status, and marital status) were analysed descriptively using means, standard deviations and frequency counts. Usability issues from the observation sessions and the feedback questionnaire were collated and an inventory was created of issues and suggestions for improvement of the app.

The team forming process was scrutinised by examining the app usage history records to determine the total number of invitations sent, number of invitations accepted, and days taken to form a team.

Usage data were descriptively analysed to determine participant retention and engagement.

To investigate the effectiveness of the app, total weekly activity time was calculated from the Active Australia Survey at baseline and 21 days. Since data were non-parametric, the Wilcoxon Mann-Whitney test was used.

## Results and Discussion

The results relating to each of the study aims are presented together with discussion in the following section, in order to assist readers to comprehend the issues and subsequent actions that were taken during the study.

### Usability of the app

Direct observation of participants using the app for the first time revealed that they were able to successfully navigate the app and undertake key tasks with no issue. Participant feedback obtained via the feedback questionnaires, indicated that the strengths of the app were its ease of use and navigation; that participants enjoyed being part of a team, and that they liked the ability to monitor their own and their team mates’ progress. The key usability issues were related to access of the app using different devices, operating systems and software. Participants reported difficulty finding the app on Apple devices (iPhone/iPad), as well as seeing all features on a Smart phone screen due to the small screen size. Variability in different users’ security settings and operating software were also found to affect some features of the app (e.g. sending invitations during the team-forming process).

Midway through the usability study, the issue of navigating to the app on mobile devices was addressed by providing a web link and instructions for Smart phone and iDevice users in all emails generated by the app including weekly emails (summarising step achievements and reminding participant to log their steps), and emails received when messages are posted on the message wall or when a gift is received. A “help tab” and additional instructions were also included to assist users to troubleshoot if they have difficulty with features due to security settings or operating software. These changes resolved the difficulties during the remainder of the usability study.

### Team forming process

Ten women were invited to be “Captains”, of whom six expressed willingness to participate in the first face-to-face session. Each of these six women sent invitations to their Facebook friends to form respective teams, however only five teams proceeded. The sixth “Captain” was highly physically active, and reported difficulty recruiting team mates who were interested in participating in a pilot study related to physical activity, as her friends were also highly active. Since we had originally only planned to recruit five “Captains” to the study, it was decided not to replace this participant and to include five captains. The participation rate for “Team Members” was 68.4 per cent (percentage of women who accepted invitations; note that this includes invitations sent by the sixth “Captain” who did not successfully form a team). A summary of the team forming process is provided in Table 1.

**Table 1:** Shows team formations including the number invitations sent, the number of invitees who declined or didn’t respond to invitations for each team, the number of day to reach minimal team numbers and the number of days to finalise the team.

Team	No. of invitations sent	No. who declined or didn’t respond to invite	No. of days to reach minimal team number (4)	No. of days to finalise the team
1	8	3	2.5	13
2	6	2	7	12
3	7	1	1	7
4	5	1	1	18
5	5	0	1	21
6	7	5	Didn’t form a team	Didn’t form a team

Teams took a median of 13 days to form. During the pilot process it became apparent that team formation was impeded by three key issues: 1) some invitees downloaded the app but did not realise that they also needed to complete a registration page (within the app) to finalise their registration; 2) the app initially did not allow participants to formally decline an invitation, causing participants who did not want to participate to appear in limbo in the system; 3) “Captains” were reluctant to finalise their teams when there were friends who had not responded, even if they had met minimum numbers to form a team.

**Figure 4:**
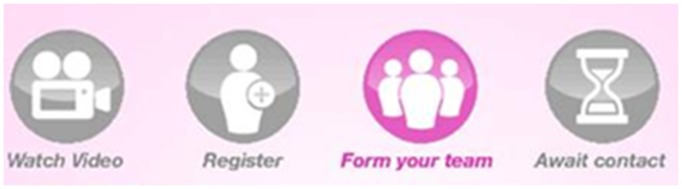
Step-by-step graphic added to the app to aid with participant registration.

These issues were dealt with midway through the pilot period. Two teams (11 participants) were recruited following these changes which enabled further testing. A step-by-step graphic (Figure 4) was added to visually highlight the process involved in online registration. A “decline invitation” function was also added, which, rather than keeping non-participants in a limbo status, cleared them from the system, allowing “Captains” to send invitations to new potential participants. Since completion of the usability study, issue three was dealt with by providing “Captains” with written guidance on appropriate time frames (i.e. the team forming screen of the app, as well as the team-forming daily email update recommends they only wait a maximum of 7 days for their friends to respond before finalising their team).

One unexpected result was that during the study, a team which did not include women who had been specifically recruited for the study, formed on its own. This team was not included in the study as recruitment had already been finalised. It is likely that the “Captain” of this unexpected team saw the Mums Step It Up invitation on a friend’s Facebook page, demonstrating preliminary evidence of the potential viral capabilities of the intervention.

### Effectiveness of the Mums Step It Up app

A total of 29 participants (5 “Captains” and 24 “Team Members”) joined the study of which 25 completed (5 “Captains” and 21 “Team Members”) (Table 2). Reasons for withdrawal included: major illness (n = 1), going overseas for an extended period (n = 1) and other commitments (n = 2). Due to missing data these four women were not included in the analysis. However, all participants who provided 21 day follow up data were included in the effectiveness analyses, irrespective of intervention compliance.

**Table 2:** Summarises the demographic characteristics of participants who completed and dropped out of the study.

Participants completing (n = 25)		Participants dropped out (n = 4)
Mean age	34.3 (2.9)	32 (5.0)
**Number of children**		
1 child	9	3
2 children	13	1
3 children	3	0
**Education**		
Some high school	0	2
Complete year 12	2	1
Diploma/TAFE certificate	2	0
University Degree or higher	21	1
**Work Status**		
Not working or on maternity leave	8	4
Working part-time	16	0
Working full-time	1	0
**Marital Status**		
Married	19	3
Defacto relationship	5	1
Single	1	0

Number of participants in each of the categories is described, with the exception of age which is presented as mean (SD).

Participants’ physical activity levels at baseline and 21 days were compared using Wilcoxon Mann-Whitney test. Participants’ physical activity significantly improved across the intervention period from a mean (SD) of 294 (307) minutes per week at baseline, to 471 (437) minutes in the final week of the program (*p* = 0.01). The number of physical activity sessions per week and the mean minutes per week increased in each of the activity categories. At both time points, walking was the most popular physical activity.

### Participant engagement and retention

Usage data recorded by the Mums Step It Up app is summarised in Table 3. In general, usage rates were high. Of the 25 participants who completed assessments at both time points 18 logged steps for all 28 days; 22 participants logged steps for more than 21 days; and 24 logged steps for more than 14 days. The minimum number of days steps were logged was 13.

**Table 3:** Shows usage data recorded by the Mums Step It Up app.

App usage data	Week 1	Week 2	Week 3	Week 4	Total
No. of logins	4.0 (1.2)	3.5 (2)	3.0 (2)	2.9 (2.1)	13.6 (6.2)
Days steps logged	6.9 (0.3)	6.8 (0.8)	6.4 (1.4)	5.5 (2.8)	25.6 (4.5)
Step count	71588 (11730)	72896 (20772)	66446 (16774)	52798 (27497)	263741 (59510.1)
Wall posts	2.2 (1.8)	1.0 (1.2)	0.5 (0.8)	1.1 (1.4)	4.8 (4.0)
Gifts sent	0.6 (1.2)	1.0 (1.7)	0.3 (0.6)	0.6 (1.1)	2.5 (2.8)

Data are presented as mean (SD).

The mean number of weekly step entries (ranging from 6.9 (SD 0.3) to 5.5 (SD 2.8)) was high compared to other social networking interventions which have included logging of physical activity [16] (maximum mean entries per week over 12 weeks was 2.1 (SD 3.4)) or dietary behaviour [29] (maximum entries per week over 6 months was 2.9 (SD 1.9)).

A modest decline in log in rates (the number of logins, numbers of days for which steps were logged and number of steps taken) was observed towards the end of the 28 day period, which is consistent with other web-based physical activity interventions [30].

The attrition rate for this study was 14 per cent, showing promise compared to other web-delivered physical activity interventions (average attrition of 27%) [30]. However, it must be acknowledged that the intervention period in this study was relatively short and attrition would likely increase over a longer time frame.

As expected, logging steps was the most used feature. Sending gifts wasn’t well utilized, and feedback by participants at the end of the program suggested that this may have been due to lack of clarity about the gifts and/or the limited range of gifts available. This has since been addressed by increasing the range of gifts available (new gifts are released during the program as participants reach particularly step count milestones) and a short description of the gift (e.g. “sent flowers for encouragement”) appears when users hover their mouse over the gift.

## Conclusion

This study aimed to assess the usability and pilot the Mums Step It Up app, a new social networking physical activity intervention for women with young children. The key findings highlighted that women with young children found the Mums Step It Up app engaging (with high usage rates and weekly step entries). The online team-building process which is a unique feature of the app appears to be feasible. In addition, the study provides preliminary evidence that a social networking intervention is effective in bringing about behaviour change.

The technical challenges of delivering a physical activity intervention in the Facebook environment where a wide variety of devices, operating systems and software platforms are in use, was highlighted. Given that these technologies are rapidly evolving, addressing such issues will be an ongoing process, and presents a sizable challenge to programmers working in the online social-network environment who wish to create robust systems.

The snowballing recruitment method and self-organising nature of this intervention were strengths of this study. It is acknowledged that the small sample size, homogenous nature of the sample (largely tertiary educated, active women), lack of control group, and use of a self-reported physical activity measure (which had moderate reliability and validity; similar to other self-report physical activity surveys [31]) limit the evidence regarding the effectiveness of the Mums Step It Up program. A randomised controlled trial is needed to see if behaviour change can be replicated with a higher quality research design, and maintained over a longer period.

## Supporting Information

File S1 Feedback Questionnaire.
(PDF)

File S2 Data.
(XLSX)
