# Supplementary material for: Usability Testing and Piloting of the Mums Step It Up Program - A Team-Based Social Networking Physical Activity Intervention for Women with Young Children
Source: PLoS One. 2014 Oct 1;9(10):e108842. doi: 10.1371/journal.pone.0108842 (PMC4182756; doi:10.1371/journal.pone.0108842)
Supplement: File S1 — Feedback Questionnaire. (PDF) [file pone.0108842.s001.pdf]

Instructions for completing the survey

Thank you for agreeing to participate in the research study titled: Piloting of the Mums Step it Up Facebook app.

We would like to find out some information about your physical activity and your opinions of the Mums Step it Up Facebook app

This survey should take approximately 10-15 minutes to complete.

Your answers are completely confidential, only the Mums Step it Up research staff will see your responses. Individual responses will not be identified in the reporting of this research.

Please read all of the questions carefully and answer as accurately as you can. If you feel uncomfortable about answering any of the questions please leave them blank.

Some questions will ask you to write your response in a text box.

Other questions can be answered by ticking a box

When you have completed each page click on "next" to go to the next page. When you have finished the survey please click on "done" and your survey will be submitted.

***1. Please enter the ID number that was provided in the email. (Please refer back to the email which provided you with the link to this survey).**

Feedback Questionnaire

We would like to get your feedback about the Mums Step it Up app.

Please complete the questions below by clicking on the number that most applies to how you feel. Space has also been provided for you to write any comments or suggestions which may assist us to further refine the app.

If you feel uncomfortable about answering any of the questions please leave them blank

2. The Facebook app is easy to use:

◯ 1 strongly disagree ◯ 2 ◯ 3 ◯ 4 ◯ 5 strongly agree

Comment

3. In my opinion the Facebook app is of interest to women who have recently had a baby.

◯ 1 strongly disagree ◯ 2 ◯ 3 ◯ 4 ◯ 5 strongly agree

Comment

4. The language used in the Facebook app is easy to follow

◯ 1 strongly disagree ◯ 2 ◯ 3 ◯ 4 ◯ 5 strongly agree

Comment

5. I have enjoyed using the Facebook app.

◯ 1 strongly disagree ◯ 2 ◯ 3 ◯ 4 ◯ 5 strongly agree

Comment

6. The look and features of the Facebook app are appealing.

◯ 1 strongly disagree ◯ 2 ◯ 3 ◯ 4 ◯ 5 strongly agree

Comment

7. The selection of gifts available to send to team mates is sufficient.

◯ 1 strongly disagree ◯ 2 ◯ 3 ◯ 4 ◯ 5 strongly agree

Comment

8. The awards are interesting and motivating.

◯ 1 strongly disagree ◯ 2 ◯ 3 ◯ 4 ◯ 5 strongly agree

Comment

9. The statistics on life gained, fat burned, carbon saved and travel costs are interesting and motivating.

◯ 1 strongly disagree ◯ 2 ◯ 3 ◯ 4 ◯ 5 strongly agree

Comment

10. The app has provided me with enough feedback about the steps that I have accrued.

◯ 1 strongly disagree ◯ 2 ◯ 3 ◯ 4 ◯ 5 strongly agree

Comment

11. The app has allowed me to compare my progress, with my team mates.

◯ 1 strongly disagree ◯ 2 ◯ 3 ◯ 4 ◯ 5 strongly agree

Comment

12. I would recommend this Facebook app to other women who have recently had a baby

◯ 1 strongly disagree ◯ 2 ◯ 3 ◯ 4 ◯ 5 strongly agree

Comment

13. The Facebook app has helped me to interact with my team members.

◯ 1 strongly disagree ◯ 2 ◯ 3 ◯ 4 ◯ 5 strongly agree

Comment

14. Logging my step counts on a daily basis is easy

- ◯ 1 strongly disagree
- ◯ 2
- ◯ 3
- ◯ 4
- ◯ 5 strongly agree

Comment

15. The pedometer is easy to use

- ◯ 1 strongly disagree
- ◯ 2
- ◯ 3
- ◯ 4
- ◯ 5 strongly agree

Comment

16. The Facebook app has helped me to increase my physical activity

- ◯ 1 strongly disagree
- ◯ 2
- ◯ 3
- ◯ 4
- ◯ 5 strongly agree

Comment

17. The Facebook app has helped to promote competition

- ◯ 1 strongly disagree
- ◯ 2
- ◯ 3
- ◯ 4
- ◯ 5 agree

Comment

18. The pedometer has helped me to increase my physical activity

- ◯ 1 strongly disagree
- ◯ 2
- ◯ 3
- ◯ 4
- ◯ 5 strongly agree

Comment

19. My team mates have been supportive

◯ 1 strongly disagree ◯ 2 ◯ 3 ◯ 4 ◯ 5 strongly agree

Comment

20. Being part of a team has helped me to increase my physical activity

◯ 1 strongly disagree ◯ 2 ◯ 3 ◯ 4 ◯ 5 strongly agree

Comment

21. Having a team captain has been helpful during the Mums Step it Up Challenge

◯ 1 strongly disagree ◯ 2 ◯ 3 ◯ 4 ◯ 5 strongly agree

Comments

22. How has your team captain assisted you during the challenge

23. What were the 3 best things about the Facebook app?

1

2

3

24. What were the 3 worst things about the Facebook app?

1

2

3

25. What changes would you suggest to help improve the Facebook app?
